# Cycling and Pedestrian Injuries in the Region of Peel, Ontario; An application of linked administrative health data to enhance local road safety and health service delivery, through an Applied Health Research Question (AHRQ)

**DOI:** 10.23889/ijpds.v9i5.2799

**Published:** 2024-09-18

**Authors:** Natalie Troke, Lesley Plumptre, Refik Saskin, Diana An, Natasha Fortin

**Affiliations:** 1 ICES

## Objectives

An Applied Health Research Question request from Peel Public Health was investigated, to provide a comprehensive overview of injury patterns and healthcare utilization, among cyclists and pedestrians in the Peel Region of Ontario.

## Approach

Emergency department visits and hospitalizations across the Peel region were analyzed from 2018 to 2022, and categorized by collision type (pedestrian, cyclist motor vehicle collision (MVC), and cyclist non-MVC). Data was segmented quarterly and annually, and further stratified by residence status, sex, and age, by linking individuals to other administrative health databases. Additionally, 30-day mortality outcomes were evaluated. To contextualize incidence rates with population demographics, Public Health Data Zone and Census Subdivision data were also utilized.

## Results

For ambulatory visits, a substantial majority of Peel residents received treatment within the region, evidenced by 4,483 cyclist visits (55%) and 1,338 pedestrian visits (50%). However, a noteworthy portion of Peel residents — 188 cyclists (45%) and 182 pedestrians (46%) — received treatment outside the region. Hospitalization patterns echoed these visits. Overall, non-MVCs accounted for a large proportion of cycling incidents, particularly among females, stressing the need for targeted safety measures. Still, survival rates post-incident were notable, with a 100% survival rate for cyclists after ambulatory visits. Pedestrian survival rates were similarly high.

## Conclusion

This study provided valuable insights into patterns of pedestrian and cyclist injuries among Peel residents, revealing considerable cross-regional healthcare utilization.

## Implications

Findings will be utilized by the Region of Peel and partner organizations, to enhance local road safety initiatives and service delivery for vulnerable road users.

